# 3D-Printing of Arteriovenous Malformations for Radiosurgical Treatment: Pushing Anatomy Understanding to Real Boundaries

**DOI:** 10.7759/cureus.594

**Published:** 2016-04-29

**Authors:** Alfredo Conti, Antonio Pontoriero, Giuseppe Iatì, Daniele Marino, Domenico La Torre, Sergio Vinci, Antonino Germanò, Stefano Pergolizzi, Francesco, Tomasello

**Affiliations:** 1 Department of Neurological Surgery, University of Messina; 2 Radiation Oncology, University of Messina; 3 Neuroradiology, University of Messina; 4 Neurosurgery, University of Messina

**Keywords:** 3d printing, arteriovenous malformation, vascular modeling, frameless radiosurgery, Stereotactic Radiosurgery

## Abstract

Radiosurgery of arteriovenous malformations (AVMs) is a challenging procedure. Accuracy of target volume contouring is one major issue to achieve AVM obliteration while avoiding disastrous complications due to suboptimal treatment. We describe a technique to improve the understanding of the complex AVM angioarchitecture by 3D prototyping of individual lesions.

Arteriovenous malformations of ten patients were prototyped by 3D printing using 3D rotational angiography (3DRA) as a template. A target volume was obtained using the 3DRA; a second volume was obtained, without awareness of the first volume, using 3DRA and the 3D-printed model. The two volumes were superimposed and the conjoint and disjoint volumes were measured. We also calculated the time needed to perform contouring and assessed the confidence of the surgeons in the definition of the target volumes using a six-point scale.

The time required for the contouring of the target lesion was shorter when the surgeons used the 3D-printed model of the AVM (p=0.001). The average volume contoured without the 3D model was 5.6 ± 3 mL whereas it was 5.2 ± 2.9 mL with the 3D-printed model (p=0.003). The 3D prototypes proved to be spatially reliable. Surgeons were absolutely confident or very confident in all cases that the volume contoured using the 3D-printed model was plausible and corresponded to the real boundaries of the lesion. The total cost for each case was 50 euros whereas the cost of the 3D printer was 1600 euros.

3D prototyping of AVMs is a simple, affordable, and spatially reliable procedure that can be beneficial for radiosurgery treatment planning. According to our preliminary data, individual prototyping of the brain circulation provides an intuitive comprehension of the 3D anatomy of the lesion that can be rapidly and reliably translated into the target volume.

## Introduction

Stereotactic radiosurgery (SRS) can induce the obliteration of cerebral arteriovenous malformations (AVMs) within 24–48 months, in up to 90% of treated patients [1-3]. However, several factors will influence the outcome of stereotactic radiosurgery (SRS), including the volume, prescription dose, patient age, lesion location, angioarchitecture, and previous embolization [1-6]. Accuracy in delineating the target volume is a major issue for their successful treatment [2, 7]. In fact, the so-called “nidus” of the AVM, a tangle of malformed blood vessels, should be entirely included in the target volume to achieve obliteration of the lesion. Furthermore, because high radiation doses are necessary to result in AVM resolution, highly conformal dose distributions should be achieved to reduce, as much as possible, the risk of radiation-induced brain complications.

Biplanar digitally subtracted angiography (DSA) is the benchmark imaging modality to delineate cerebral AVMs in SRS treatment planning. At present, DSA is the only imaging study that can provide sufficient temporal resolution to study the AVM hemodynamics [8]. It also provides the greatest spatial resolution. Nevertheless, when using DSA, the target volume is reconstructed from the contours drawn independently on two or more perpendicular or oblique two-dimensional (2D) views, using stereo-viewing conditions. Because of the bidimensional nature of DSA, the three-dimensional (3D) target volume cannot be reconstructed with complete accuracy [9]. This limitation of DSA has long been acknowledged, especially in the case of complexly shaped AVMs [10].

The CyberKnife (Accuray Inc., Sunnyvale, CA) is a frameless system for SRS. It uses noninvasive image-guided localization, a lightweight high-energy radiation source, and a robotic delivery system to deliver SRS in single or multiple sessions [11-19]. With the CyberKnife, the reference coordinates for the intracranial space are not provided by a stereotactic frame but are obtained directly from the skull. A skull-tracking system compares the pretreatment computed tomography (CT) images with images of the skull obtained during treatment delivery. Accordingly, the use of biplanar stereotactic DSA is not available for this frameless SRS system. Instead, volumetric imaging, such as 3D-CT angiography (CTA) and 3D magnetic resonance (MR) angiography (MRA), are used. Both CTA and MRA improve the 3D target volume definition, but they can lack sufficient spatial and temporal resolution to discriminate among the nidus, normal vessels, such as the feeding arteries or draining veins, and the immediately surrounding normal vessels.

Three-dimensional rotational angiography (3DRA) is a volumetric angiographic study generated from a rotational sequence of biplanar DSA images. Because of its planar reconstruction in Digital Imaging and Communications in Medicine (DICOM) format, it can be integrated in CyberKnife treatment planning. This imaging modality appears very effective in improving three-dimensional interpretation of the target volume and the following treatment. Since we reported the value of this imaging modality for the treatment of AVMs [16], the treatment planning systems have evolved offering a streamlined integration of this imaging modality and the possibility of performing the contouring of AVMs using the impressively detailed 3D reconstructions of the 3DRA. Nevertheless, AVMs are very complex lesions and a satisfactory 3D volume rendering does not always allow the generation of a precise mental image of the target volume. This is even more complicated when the AVM is partially embolized and spread out in multiple parts whose anatomy is not always intuitive.

In light of these difficulties and the necessity to precisely define the target to avoid disastrous complications, including brain radionecrosis or bleeding from a residual portion of the AVM, we have attempted to achieve an improved interpretation of each AVM's anatomy by printing 3D prototypes of individual patient AVMs. Our findings will be presented in this paper.

## Materials and methods

### Patients

Neuroimaging data of ten patients with AVMs who underwent SRS at the CyberKnife Center of the University of Messina, Italy, between January 2014 and January 2016 were selected for re-review and treatment planning. We selected cases in which the identification of the target volume was not intuitive. Difficulties were due to the superimposition of different vascular components such as: giant draining veins, arterial and venous aneurysms, normal vessels superimposed upon the AVM, convoluted and hypertrophic arterial feeders, neo-angiogenesis phenomena surrounding the nidus, or fragmentation of the nidus due to dispersed embolizing material. These factors were differently combined in different patient cases. The procedure of SRS target contouring and treatment planning was performed using the MultiPlan® Treatment Planning System (Accuray Inc., Sunnyvale, CA). Informed consent was obtained from all patients for this study.

### Image acquisition and processing

The image acquisition process has been previously described [16]. Briefly, all patients underwent volumetric CTA performed using a multislice CT scanner (Siemens Sensation 16, Siemens, Erlangen, Germany). Magnetic resonance imaging (MRI) was obtained using a Magnetom Vision 1.5 T scanner (Siemens, Munich, Germany). A multiplanar reformatting, gradient echo, contrast-enhanced volumetric study was performed together with a 3D time of flight MR angiography acquisition sequence.

Two-dimensional angiographic examinations were carried out using a commercially available biplanar angiographic unit (Axiom Artis, Siemens, Munich, Germany). Selective four- or six-vessel angiography, using a standard projection format, was performed initially, and additional views were obtained if required to identify the parent vessel and aneurysm neck more clearly. Rotational angiography was done using the frontal plane of a biplanar C-arm. The C-arm rotates in a continuous 180° (+90° to -90°) arc with the path of the x-ray tube. The isocenter for the rotational field was the area of interest in the patient’s head. A total of 133 digital angiograms with a 512 x 512 matrix were obtained during the 180° rotation of the C-arm. The total rotation and angiogram acquisition was accomplished within eight s.

**Figure 1:**
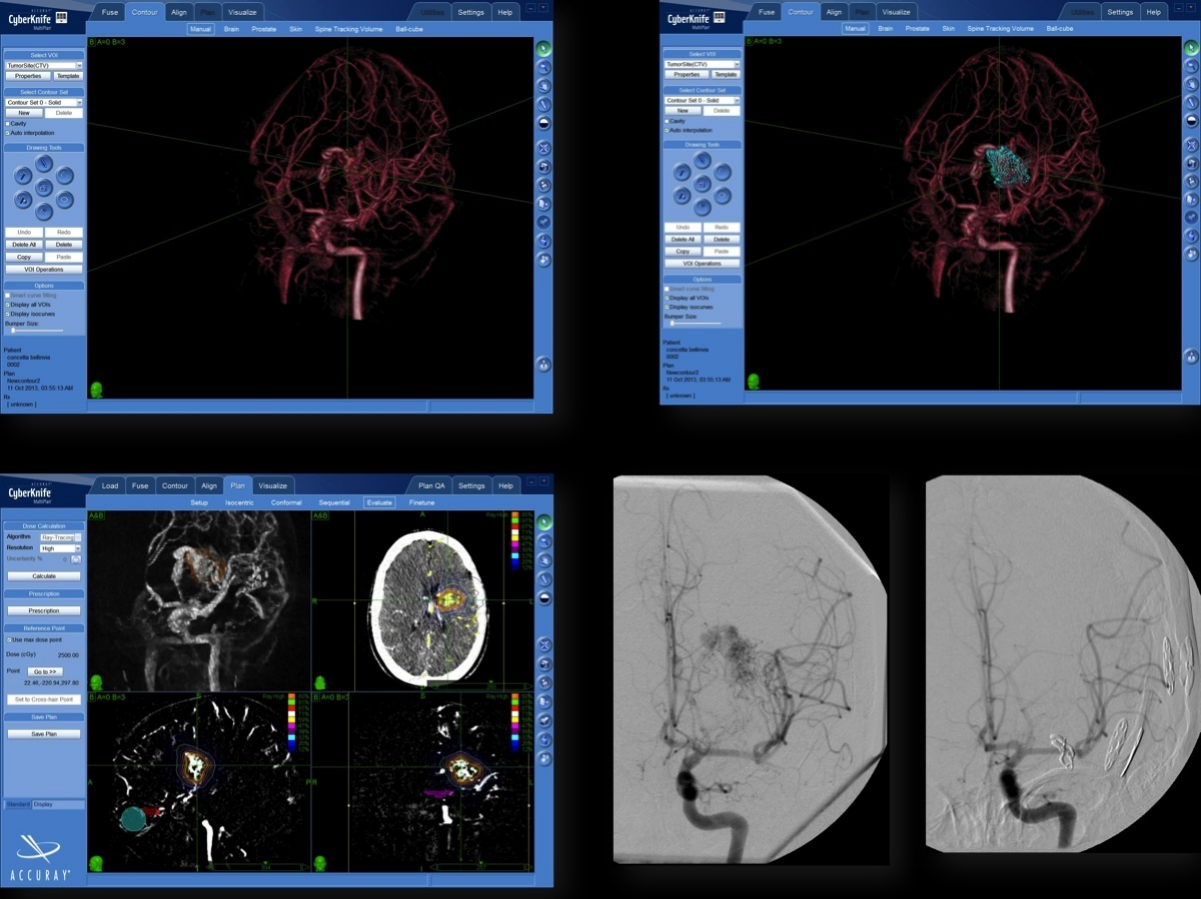
Integration of 3D rotational angiography in CyberKnife treatment planning of arteriovenous malformations. The CyberKnife MultiPlan system can use 3D rotational angiography (3DRA) for treatment planning. Using 3DRA, we achieved direct 3D visualization of the vascular structures and dose distributions. Optimal results could be achieved using this method in most cases.

The rotational sequence was used as input to a tomographic reconstruction algorithm that produced as output a 3DRA volume on a cube of 256 x 256 x 256 voxels. The resulting volume was a relatively high-resolution 3D reconstruction of the vasculature, the 3DRA. A volumetric data set, the 3D (multiplanar reformatting) full/native image set, was generated and then sent to the CyberKnife workstation. The 2D cut planes, obtained from the 3DRA image, were used as the secondary images to be coregistered with the CTA images. Subsequently, conventional 2D contouring in three planes (sagittal, coronal, and axial) was performed (Figure 1). In all cases, thin-slice contrast-enhanced, volumetric MR images were also obtained (Siemens Magnetom 1.5 T, Siemens, Munich, Germany). The treating surgeons manually outlined critical structures (i.e., brainstem, optic chiasm, optic nerves, and eyes) on the MR images using the MultiPlan software.

The contouring of the target volume was performed using the CTA, MRA, and 3DRA. To achieve real 3D contouring, the 3D visualization tool of the treatment planning system was used to perform an iterative delineation on the 2D angiographic cut planes and directly on the 3DRA. (Figure 1).

### 3D-printing procedure

The 3DRA of these patients were recalled and used for 3D printing. One prototype was then obtained for each lesion. To obtain the stereolithography (*.stl) file format necessary for 3D printing, we used the standard DICOM image files of the 3DRA. The DICOM images were processed using the OsiriX 32-bit software (Pixmeo SARL, Bernex, Switzerland) to obtain regions of interest (ROI). The ROIs were moved to the native module "surface rendering" of the same software. In this environment, we performed adjustments to improve the rendering of smaller details and to smooth the surfaces. These images were exported in “.stl” format and uploaded onto two 3D editing software: Blender 2.77 (Blender, the Netherlands) and Meshmixer 3.0 (Autodesk Inc., Berkeley, CA).

By using Blender, we first cut off the parts of the image not physically joined to the main vascular trunk, and then adjusted the image smoothing. Finally, we uploaded the images onto Meshmixer. Using this software, the peripheral and unnecessary portions of the image were further eliminated and supports for 3D printing were created. Once this process was completed, the final image exported in .stl format was printed with the Witbox 3D printer (BQ, Madrid, Spain) using a 1.75 mm polylactic acid (PLA) filament.

### Measurements

**Figure 2:**
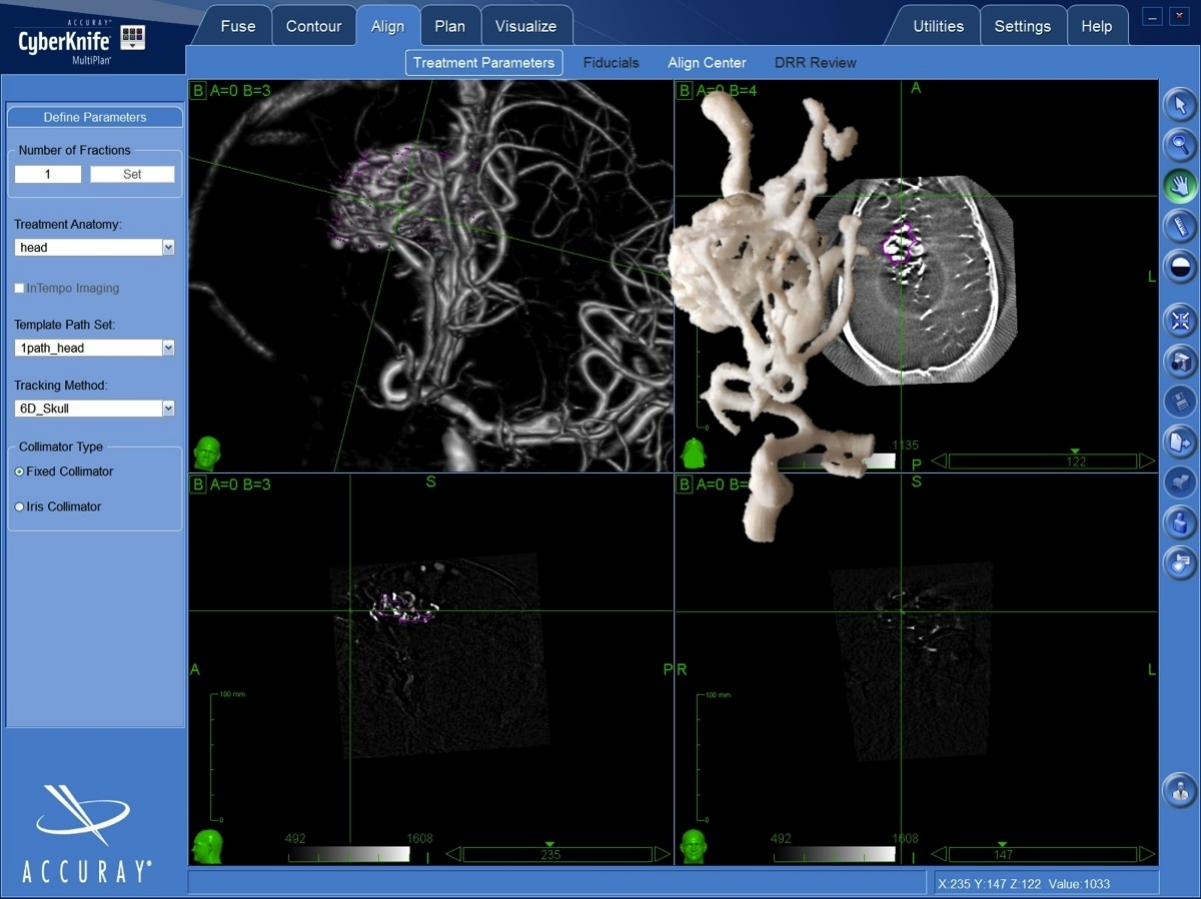
3D-printed prototypes of the arteriovenous malformations were used to help with the interpretation of the individual angioarchitecture. The figure shows the MultiPlan treatment planning system displaying the 3D rotational angiography (3DRA) volume rendering of the AVM and, superimposed, the 3D-printed prototype of the same lesion.

Two treating physicians (AC and AP) performed independently the contouring of each AVM on two different treatment planning stations. One of the two treating physicians, alternatively, used the 3D-printed prototype together with the neuroimaging (CTA, MRA, and 3DRA) to interpret the AVM volumes (Figure 2), whereas the other one used only the neuroimaging.

**Figure 3:**
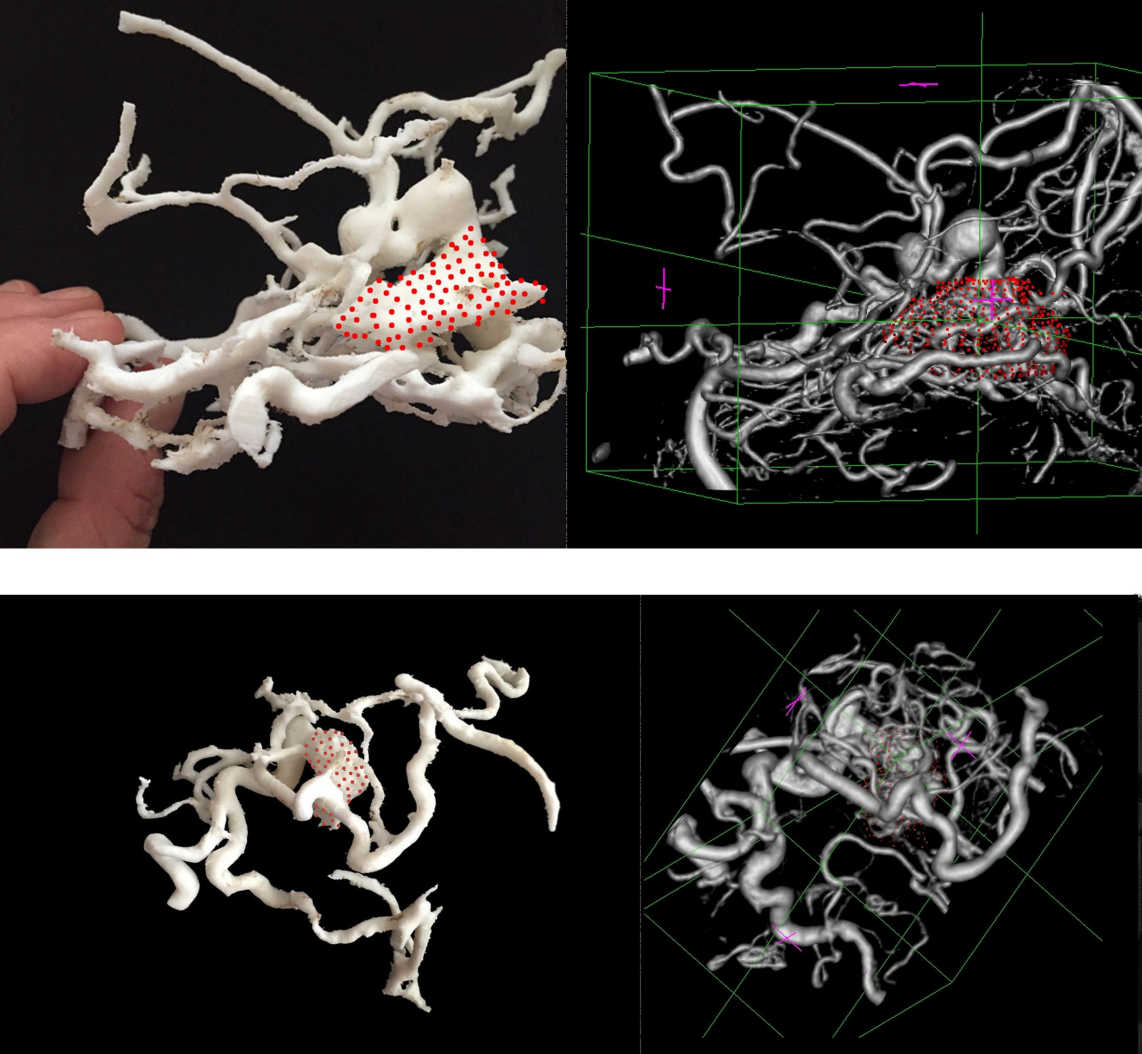
3D-printed arteriovenous malformation prototypes and the corresponding imaging. The different vascular components of the anteriovenous malformations could be easily and reliably identified in the 3D-printed models.

A qualitative and quantitative comparison of the target volumes obtained was performed. The analysis of conjoint (overlapping) and disjoint (mutually exclusive) volumes was also performed [20-21]. The analysis was carried out using the MultiPlan system tools according to the following steps: (1) a first volume was obtained from the 3DRA; (2) a second volume was generated, without knowledge of the first volume, using 3DRA and the 3D-printed model (Figure 2); (3), the two volumes were recalled and superimposed; and (4) the conjoint and disjoint volumes were measured.

Furthermore, we calculated the time needed to perform contouring and assessed the confidence of the two surgeons in the definition of the target volume using an adapted six-point scale (absolutely confident; very confident; confident; moderately confident and unconfident).

### Statistical analysis

Statistical analysis was accomplished using the Wilcoxon matched pairs test. The chi square test was used to compare contingency tables. The data analysis was performed using Instat, version 3.0, and Prism, version 4.0 (GraphPad, San Diego, CA). The values were expressed as the mean ± standard deviation.

## Results

The time required for the contouring of the target lesions was shorter when the surgeons used the 3D-printed model of the AVMs. The mean time without the 3D model was 50.2 ± 12.8 minutes whereas it was 38.9 ± 10.7 minutes with the 3D-printed model (p=0.001).

The mean volume of the target volumes was smaller when the surgeons used the 3D-printed models of the AVMs. The volume without the 3D model was 5.6 ± 3 mL, whereas it was 5.2 ± 2.9 mL with the 3D-printed model (p=0.003). The 3D prototypes were spatially reliable. Actually, vascular structures could be easily recognized on the 3D-printed prototype and we found a very precise correlation between the size of the lesion, the carotid artery, and the major draining veins as measured on the CTA or 3DRA and on the printed model (Figure 3).

**Figure 4:**
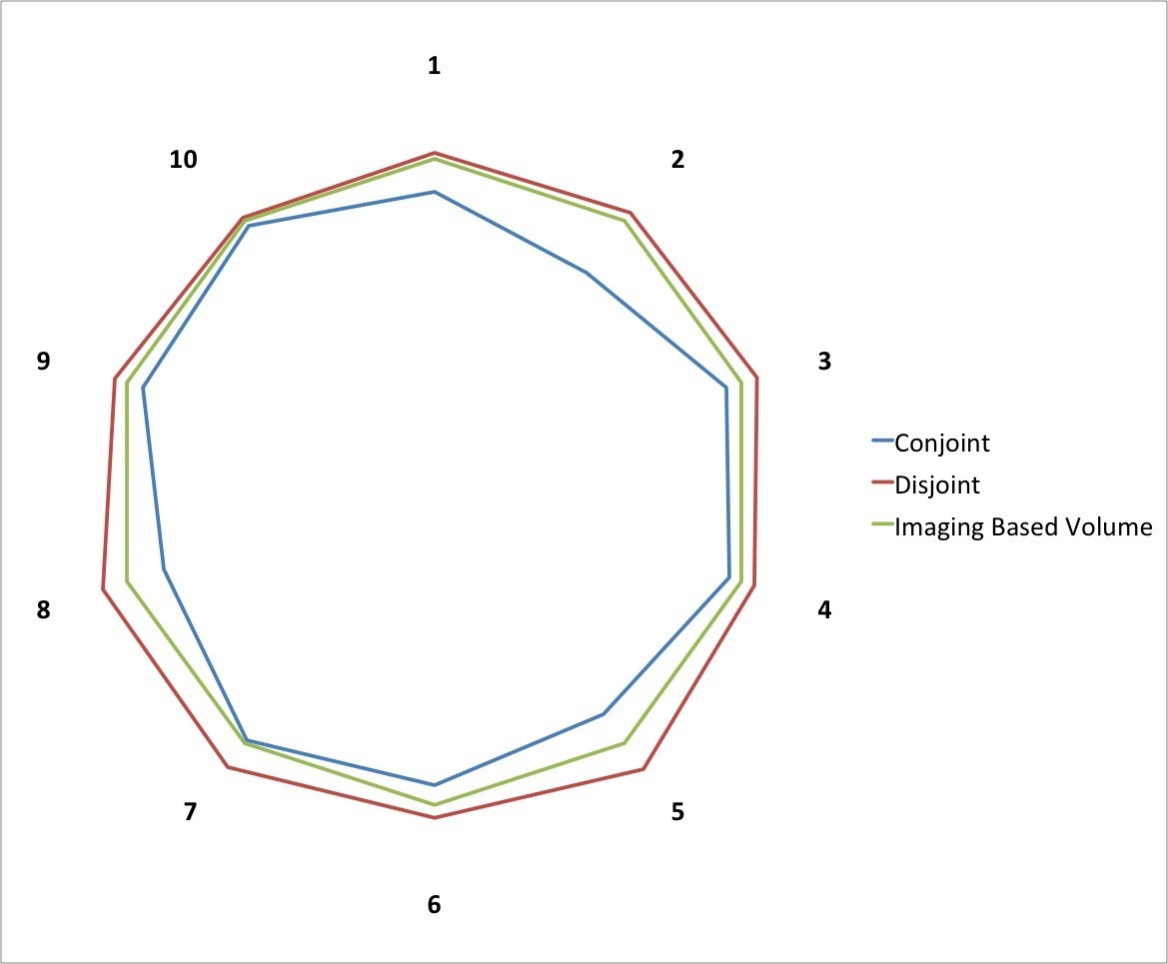
Analysis of the two volumes obtained with or without the 3D-printed model. To assess the inconsistency between the volumes obtained with or without the printed model and its magnitude, we measured the conjoint and disjoint volumes. The volume obtained using the 3D prototype was 92.4 ± 6% (blue line) of the volume obtained without the 3D model (green line). The volume obtained using the 3D-printed model included a 0.27 ± 0.1 mL that was outside the volume obtained without the aid of the 3D-printed model. The numerals 1-10 indicate individual cases.

To assess whether the volumes were coincident, we measured the conjoint and disjoint volumes [20-21]. The overlap of the two volumes was 92.4% ± 6% (Figure 4). The volume included in the target using only imaging that was not contoured using the 3D-printed model was 0.4 ± 0.02 mL (7.4% of the final volume), and the volume included using the 3D-printed model that was not present in the imaging only-based contour was 0.27 ± 0.1 mL. 

**Figure 5:**
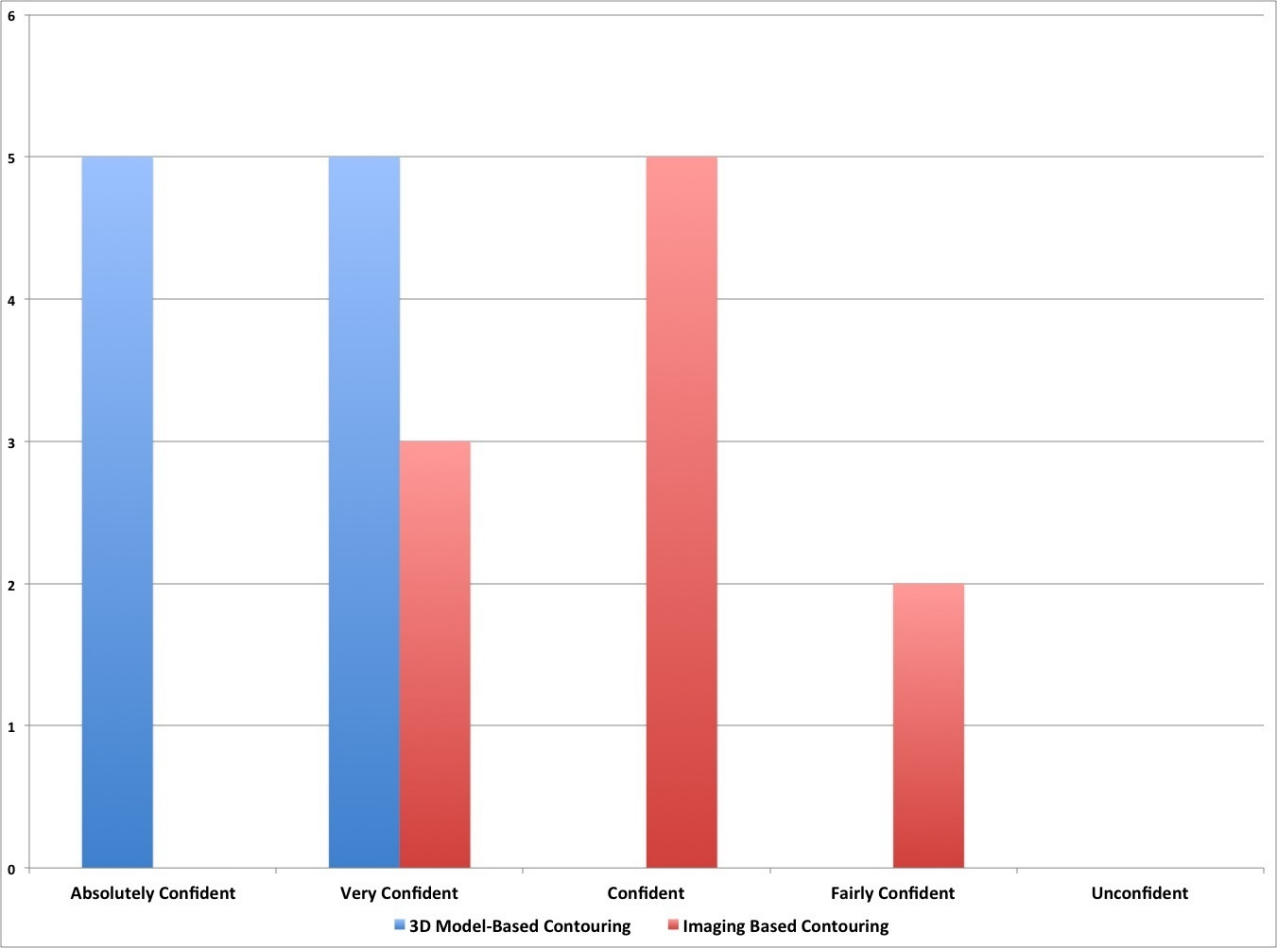
Confidence of treating physician about the target volume. We measured the confidence of surgeons about the target volume. Surgeons were absolutely confident (50%) or very confident (50%) in all cases when the volume was contoured using the 3D-printed model. The level of confidence was lower (very confident 30%, confident 50%, fairly confident 20%) when the target was contoured using only the neuroimaging. With regard to the degree of uncertainty, there was a statistically significant difference between the two groups (p=0.01).

We also measured the confidence of surgeons with regard to the final volume. Surgeons were absolutely confident (50%) or very confident (50%) in all cases that the volume contoured using the 3D-printed model was plausible and corresponded to the real boundaries of the lesion. The level of confidence was lower (very confident 30%, confident 50%, fairly confident 20%) when the target was contoured using only the neuroimaging. With regard to a certain degree of uncertainty in the procedure, there was a statistically significant difference between the two groups (p=0.01) (Figure 5).

The total cost for each case was 50 euros times 10 patients or 500 euros total, whereas the cost of the 3D printer was 1600 euros. The fabrication of each printed model, however, required between seven and 11 hours of unsupervised printing work and about 1-2 hours to remove plastic supports and to smooth the surface where supports were attached.

## Discussion

According to this initial experience, the “bedside” 3D printing of AVMs is a simple, affordable, and spatially reliable procedure that can be beneficially used for radiosurgery treatment planning. In fact, individual prototyping of the brain circulation provided an intuitive comprehension of the 3D anatomy of the target volume that could be rapidly and reliably contoured.

The identification of the correct target volume is a major issue in SRS treatment of AVMs. The vascular anatomy can be differently interpreted by physicians, which results in the contouring of different target volumes. Zhang et al. [22] compared the estimated nidus volume using conventional DSA, 3D rendering of the target volume reconstructed from bidimensional DSA, and combined CT-DSA plans. They described a significant mean nidus volume reduction of 32% using 3D-rendered DSA plans and an additional significant reduction of 10% using CT-DSA plans. The mean overlapping volume between the CT plans and CT-DSA plans was 64%. Hamm et al. [21] analyzed the data from 34 patients for whom MR imaging and MRA 3D time-of-flight data sets were used to define the target volumes. The target volumes compared with those obtained using stereotactically localized CT and DSA. The median volume was 2.14 mL^ ^for the DSA volume and 3.07 mL for the final volume. This difference was statistically significant. Accordingly, 3D stereotactic imaging is essential for conformal treatment of an AVM nidus and to preserve the surrounding normal tissue and vascular critical volumes. We used 3DRA for our treatment. Three-dimensional rotational angiography is a volumetric data set built from a rotational sequence of DSA images. This volumetric reconstruction can be segmented to obtain 2D cut planes, exactly corresponding to that of the volumetric CT scans used by CyberKnife for localization (Figure 1). After performing 3DRA, both native nonsubtracted and digitally subtracted images are available, and the bony landmarks can be used for easy and automatic co-registration of 3DRA and the CT scans for stereotactic localization. Three-dimensional rotational angiography has substantial advantages compared with 3D MRA, including greater spatial resolution and, owing to the opportunity to use bony landmarks, the potential for more precise co-registration with the localizing CT scan.

Nevertheless, AVMs are very complex lesions and a satisfactory 3D volume rendering does not always allow the generation of a precise mental image of the target volume. The nidus is often blurred by an intricate network of vascular components including: giant draining veins, arterial and venous aneurysms, normal vessels superimposed upon the AVM, convolute and hypertrophic arterial feeders and neo-angiogenesis phenomena surrounding the nidus. The precise identification of each structure is even more complicated when the AVM has been partially embolized. Such lesions are often fragmented into multiple segments whose reciprocal relationships are not always intuitive.

The correct interpretation of these complicated AVMs requires prolonged observation of the biplanar images and corresponding 3D reconstruction and, contextually, multiple switches from one imaging dataset to another (3DRA, MRA, and CTA). This eventually results in a slow conceptualization of the AVM anatomy. The ability to build a 3D construct of an object from the 2D images is part of the visual-spatial constructive cognition. Visual-spatial construction is a central cognitive ability essential for daily life. However, there are enormous individual differences among people in their ability to perform visual-spatial constructive tasks. For instances, there are individuals who can interpret complex patterns accurately and rapidly and others who can interpret only simple patterns or none at all.

With a plastic reconstruction of the object, the creation of a mental 3D image of the AVM may require much less effort. Our results support this hypothesis demonstrating that the use of a 3D prototype reduced the time and increased the confidence in the procedure of target volume contouring.

Three-dimensional printing was introduced in the manufacturing industry about a decade ago, but it is with the decreased costs and availability in very recent years that this technology has gained an increasing interest in the medical field. The cost of 3D printers has recently decreased dramatically. For instance, as of 2013, several companies and individuals are selling parts to build 3D printers with prices starting at approximately €500/US$500. This has introduced the opportunity of “bedside” 3D printing and a multitude of innovative applications. In medicine, 3D printing technology has been used to create intelligible, personalized models of the patient’s own anatomy for patient counseling, teaching, or surgical training as well as for the creation of customized prostheses and implantable medical devices [23-24].

Different applications for neurosurgery have been proposed as well. In particular 3D printing has been used for simulation of complex surgical approaches and for training of basic and advanced surgical procedures, including third ventriculostomies or spinal procedures [25-26]. Furthermore, the use of this technology for the development of customized implantable devices, i.e. cortical electrodes arrays, to treat functional disorders or epilepsy can be envisaged in the very near future.

Vascular neurosurgery deals with some of the most complex and intricately organized anatomic structures in the human body. The 3D printing of vascular structures has been reported in only a few studies. The majority of these are focused on printing models of intracranial aneurysms [27-28]. Three-dimensional printing of aneurysms can support advanced pre-surgical planning and an improved 3D understanding of the vascular anatomy. Arteriovenous malformations provide a further step in the application of 3D modeling for neurosurgical planning. As mentioned above, these lesions require a detailed comprehension of the patients' unique vascular anatomy. Streamlined and inexpensive 3D printing allowed us to visualize the objects of our attention using the hand and mind simultaneously. To date, no studies in the literature have described patient-specific rapid prototyping of AVMs. We describe herein a novel and translatable use of 3D printing and its potential application to the practice of radiosurgery.

## Conclusions

This is a preliminary experience requiring further clinical data to confirm its promises. Nevertheless, the use of 3D prototyping has unquestionable potential applications to radiosurgery, in particular the possibility of creating very sophisticated patient-specific phantoms for dosimetric measurements and treatment simulation [29]. It is conceivable that, in the near future, tumors, vascular structures and normal brain will be rapidly prototyped obtaining complex 3D models. These 3D models will be used for both improved comprehension of diseases and simulations of minimally invasive treatments, in particular stereotactic procedures including laser ablations, and stereotactic radiation therapy.
